# A Study on the Acoustic Response of Pickering Perfluoropentane Droplets in Different Media

**DOI:** 10.1021/acsomega.0c06115

**Published:** 2021-02-17

**Authors:** Ksenia Loskutova, Didrik Nimander, Isabelle Gouwy, Hongjian Chen, Morteza Ghorbani, Anna J. Svagan, Dmitry Grishenkov

**Affiliations:** † Department of Biomedical Engineering and Health Systems, KTH Royal Institute of Technology, Stockholm 14157, Sweden; ‡ Sabanci University Nanotechnology Research and Application Center, Istanbul 34956, Turkey; ¶ Department of Fibre and Polymer Technology, KTH Royal Institute of Technology, Stockholm 10044, Sweden

## Abstract

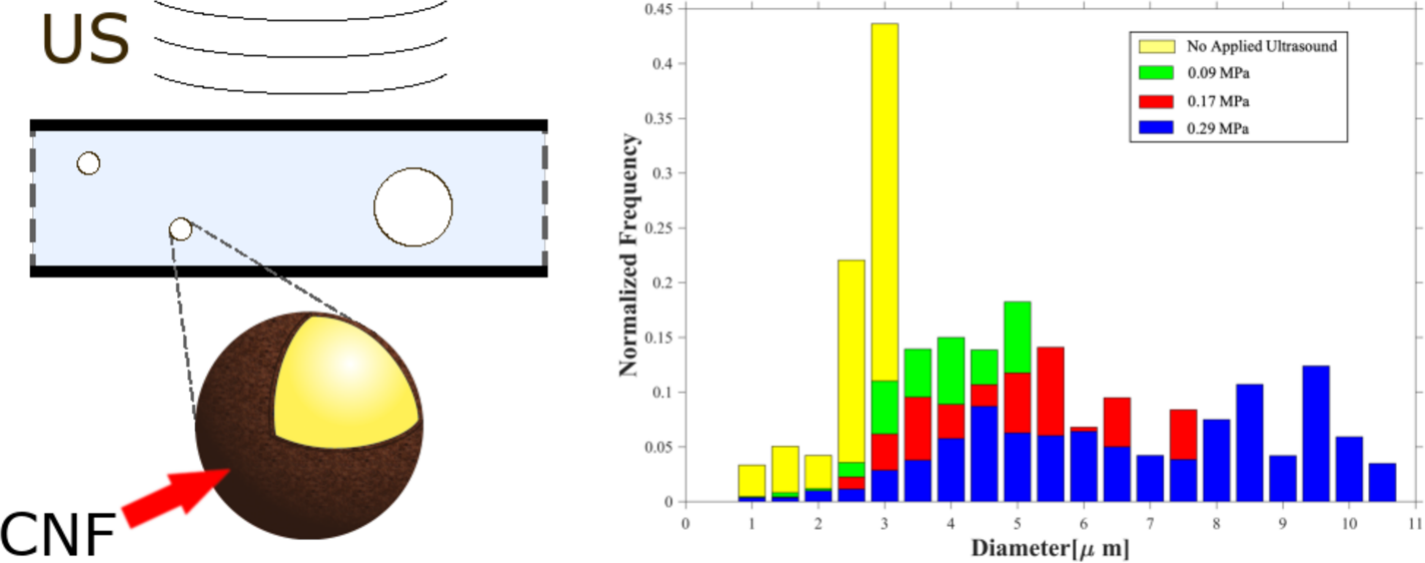

Acoustic droplet vaporization (ADV) is the physical process of liquid-to-gas phase transition mediated by pressure variations in an ultrasound field. In this study, the acoustic response of novel particle-stabilized perfluoropentane droplets was studied in bulk and confined media. The oil/water interface was stabilized by cellulose nanofibers. First, their acoustic responses under idealized conditions were examined to assess their susceptibility to undergo ADV. Second, the droplets were studied in a more realistic setting and placed in a confined medium. Lastly, an imaging setup was developed and tested on the droplets. The acoustic response could be seen when the amplitude of the peak negative pressure (PNP) was above 200 kPa, suggesting that this is the vaporization pressure threshold for these droplets. Increasing the PNP resulted in a decrease in signal intensity over time, suggesting a more destructive behavior. The imaging setup was able to differentiate between the droplets and the surrounding tissue. Results obtained within this study suggest that these droplets have potential in terms of ultrasound-mediated diagnostics and therapy.

## Introduction

In 1968, Gramiak and Shah^1^ were the first to report a contrast enhancement effect after saline injection. Ziskin et al.^2^ later concluded that this effect was due to air bubbles present in the blood stream introduced at the injection site. Since then, gas-filled bubbles have been developed and have become a vital part of contrast-enhanced ultrasound imaging. There are many commercially available gas-filled bubbles that are exploited as contrast agents, such as SonoVue and Optison, that are widely used for imaging of the heart, blood vessels, kidneys, and liver.^3,4^ These contrast agents can provide valuable medical information that is not available with conventional ultrasound imaging.

Contrast agents based on perfluorocarbons open up new possibilities in medical applications. The low solubility and low diffusivity of perfluorocarbons make the shelf life and the circulation life of phase-changing contrast agents (PCCAs) significantly longer than those of clinically available microbubbles, ranging from a few days to a couple of months.^3,5,6^ Additionally, and most importantly, perfluorocarbons are biocompatible and non-toxic.^7^ Perfluoropentane (PFC5) in particular has been used in several previous studies.^8−10^

It is worth mentioning that drugs administered intravenously to a patient are often in liquid form. Mixing two different liquids is easier and more practical than mixing a liquid with gas. Therefore, PCCAs can be considered as a strong candidate for efficient and targeted drug delivery. Additionally, through the vascular system, it is possible to reach almost any area of the body and target it locally using tissue-specific ligands. Phase changing droplets also have other promising areas of application such as embolotherapy,^11^ contrast-enhanced ultrasound imaging,^12^ theranostics,^13^ thermal therapy,^14^ and histotripsy.^15,16^

The PCCA can be converted from a liquid-filled droplet to a gas-filled microbubble by phase transition of the core by means of ultrasound exposure above a certain peak negative pressure (PNP) amplitude threshold, a process known as acoustic droplet vaporization (ADV).^17^ The vaporization occurs due to mechanical factors when the acoustic field interacts with the dispersed medium. When the droplets undergo the transformation into bubbles, they grow in size with a factor of approximately 5–6.^18^ Furthermore, in a way similar to regular microbubbles, sufficient acoustic pressure amplitude of the incident acoustic field can cause the newly formed bubbles to cavitate and burst, thus releasing the drug they carry.^7^

The dispersed medium is stabilized with surfactant molecules as a shell around the PCCA droplets. Different shell materials and their effects on the acoustic performance on PCCAs have been previously studied.^15^ Common materials are phospholipids,^19^ fluorosurfactants,^20^ and proteins.^15,21^ However, a problem that many droplets have encountered is that either their stability is compromised^16^ or the vaporization pressure threshold exceeds those that are allowed to be used in medical settings.^22^

On the other hand, by preparing Pickering PCCAs, that is, PCCAs with interfaces that are stabilized with particles, the stability can be significantly increased due to the high particle desorption energies from the PCCA/water interface and steric hindrance presented by the particles.^23^ We recently, for the first time, experimentally demonstrated that cellulose nanofibers (CNFs) are capable of stabilizing the PFC5/water interface in this way.^9^ The use of CNF is advantageous because not only do the droplets have a high stability, but the encapsulated liquid is also able to be converted into gas at clinically approved pressure amplitudes. The CNFs are also rich in hydroxyl groups, making it easy to modify the surface to accommodate ligands for tissue-specific targeted imaging and therapy.^24^ However, the effects of the confined medium on these droplets, shown to have effects on the vaporization threshold of similar droplets,^25^ are still unknown. This knowledge is important for potential clinical applications as the droplets will be confined within blood vessels.

**Figure 1:**
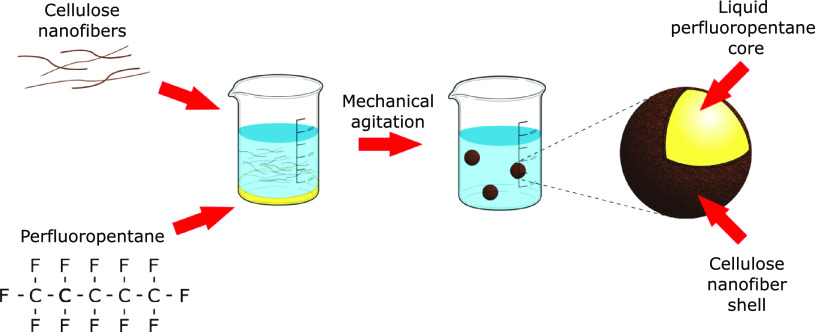
Scheme of the preparation of the CNF-stabilized PFC5-droplets.

**Figure 2:**
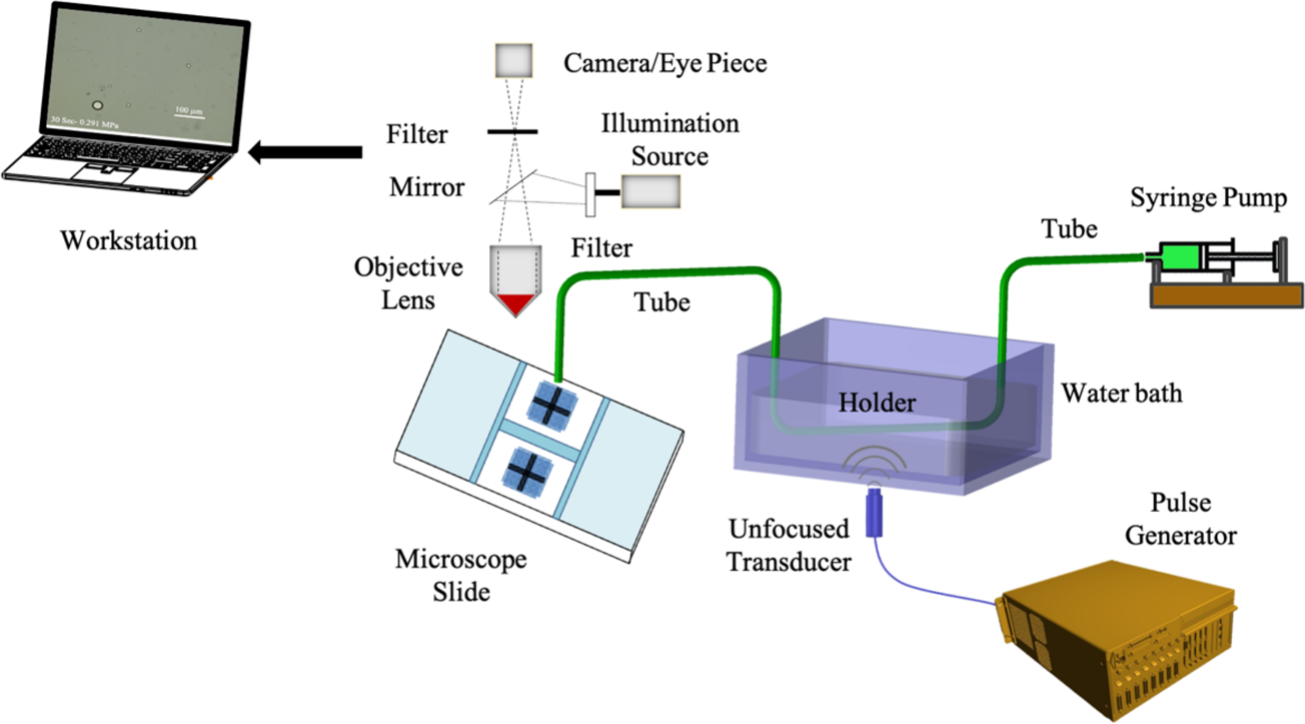
Schematic of the experimental setup for measurements with a single crystal in a confined medium.

## Results

In the present work, the interface of the PFC5 droplets was stabilized by a Pickering mechanism as demonstrated in a previous study.^9^ The advantage with CNFs is that the size of the droplets could easily be controlled by varying the amount of CNF added.^26^ Also, the resulting droplets are stable and not prone to coalescence and Ostwald ripening due to amongst other, the steric hindrance presented by the CNFs.^9^

**Figure 3:**
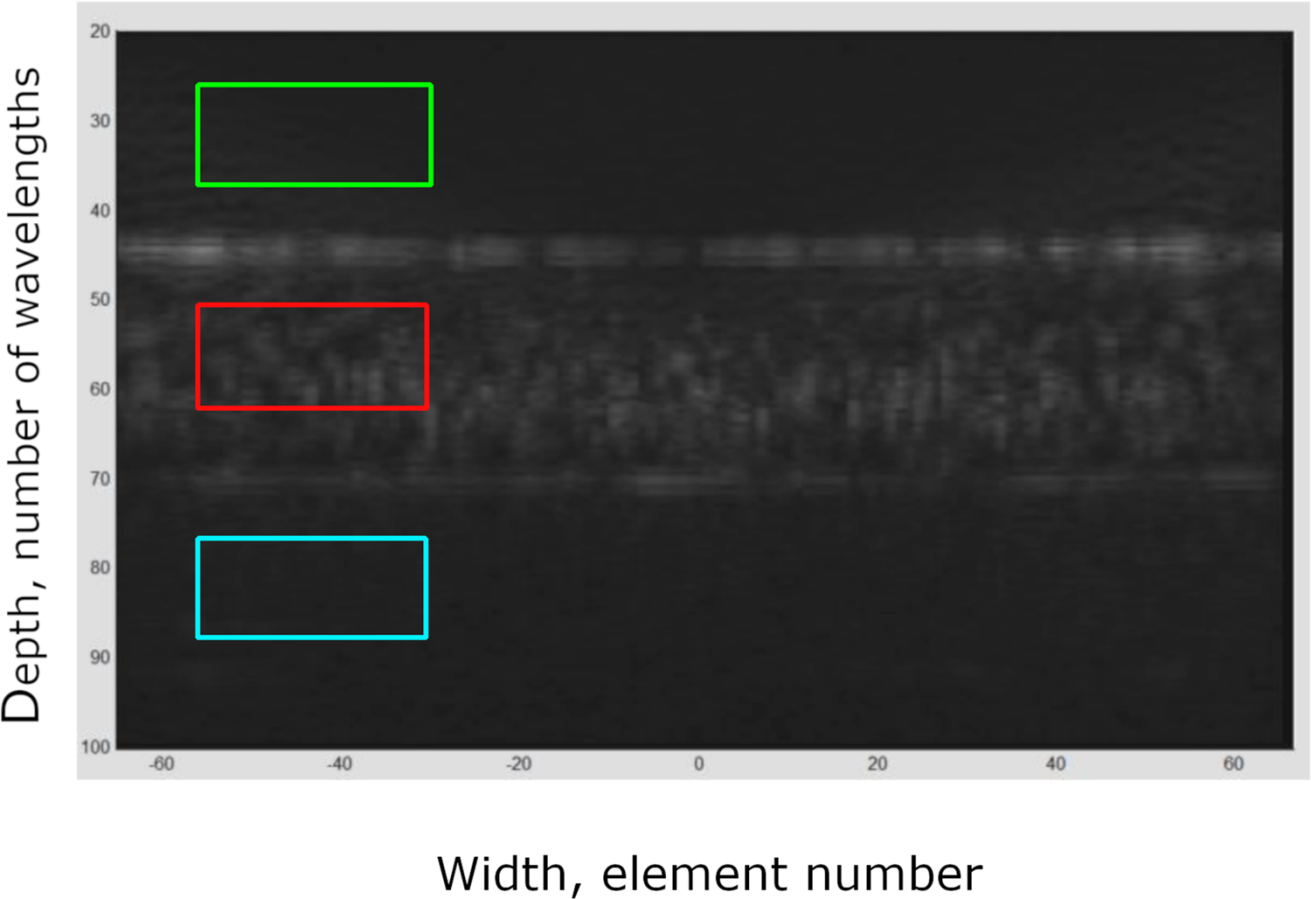
Three regions of interest (ROIs) selected for processing when studying the non-linear response of vaporized droplets in a confined medium. The area marked with red is within the vessel containing the droplet suspension, while the green and the blue areas are outside the vessel and are used for reference. One wavelength equals 0.43 mm.

### Acoustic Tests in Ideal Conditions

**Figure 4:**
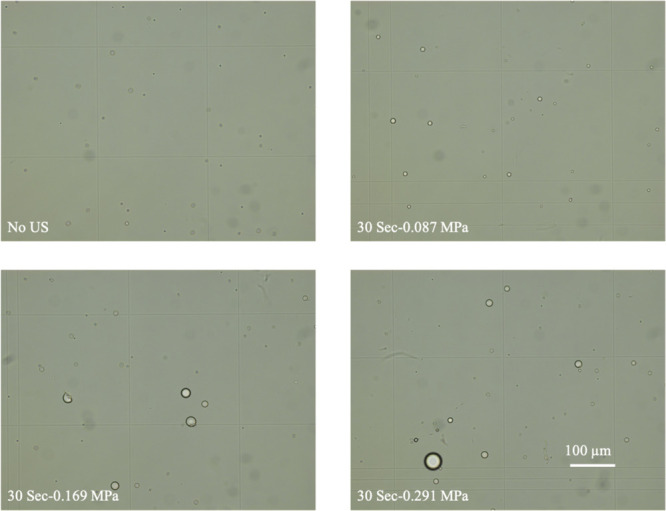
Variations in droplet volume distribution for the unfocused ultrasound at the frequency of 3.5 MHz and PNP of 0, 0.09, 0.17, and 0.29 MPa in the bulk medium–single crystal measurement. The number of larger droplets (diameter >10 μm) increases significantly as PNP increases above vaporization pressure threshold.

**Figure 5:**
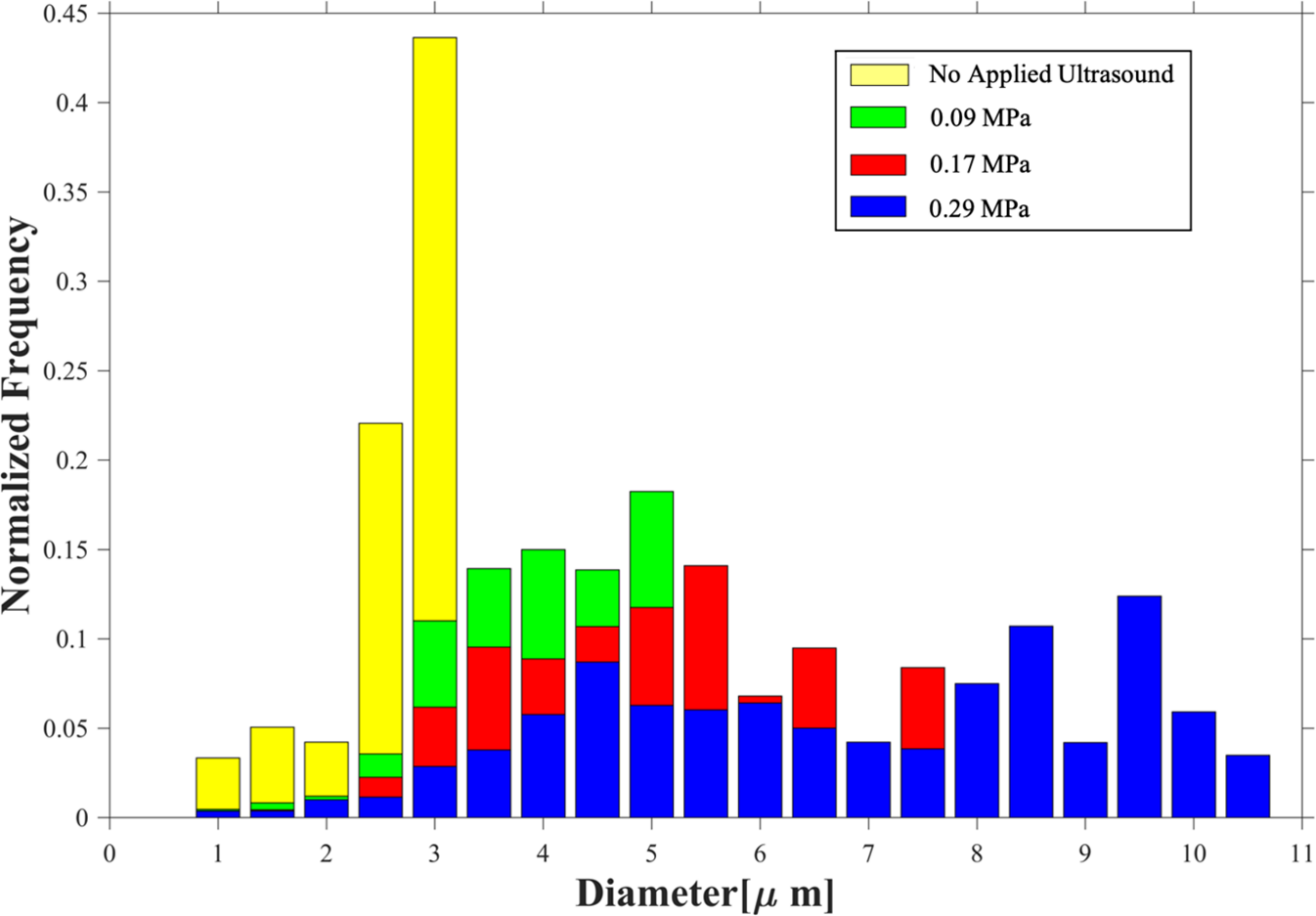
Volume distributions of CNF-stabilized PFC5 droplets at (a) no applied ultrasound, (b) 0.09, (c) 0.17, and (d) 0.29 MPa at a frequency of 3.5 MHz at a PRF of 500 Hz to study the linear response of the droplets in ideal conditions.

Figure 4 demonstrates major transitions in the intervals at 0.09, 0.17, and 0.29 MPa. According to the results in Figure 4, there is an appreciable rise of the mean volume of the droplets after ultrasound wave exposure, particularly at 0.17 MPa, similar to a previous study.^9^ As shown, the droplet mean volume increased with the pressure increase, and more bubbles with bigger volumes appeared at higher PNP. The volume distribution of the droplets at various acoustic pressures is also demonstrated in Figure 5. The number of detected droplets at 0, 0.09, 0.17, and 0.29 MPa were 1212, 912, 178, and 154, respectively. The reason for the decrease in the number of detected droplets was that vaporization took place and the converted bubbles left the focus region during microscopy imaging. As the PNP increased, the volume distribution shifted toward higher droplet diameters, and an increasing number of bubbles with a diameter above 10 μm were detected. This outcome implies the significant role of the applied PNP on the phase shift and subsequent mechanisms as a result of the acoustic wave exposure on the present droplet type.

### Effect of Confined Volume on the Acoustic Response

The acoustic behavior of the droplets in a confined medium was investigated as well as their potential as PCCAs.

**Figure 6:**
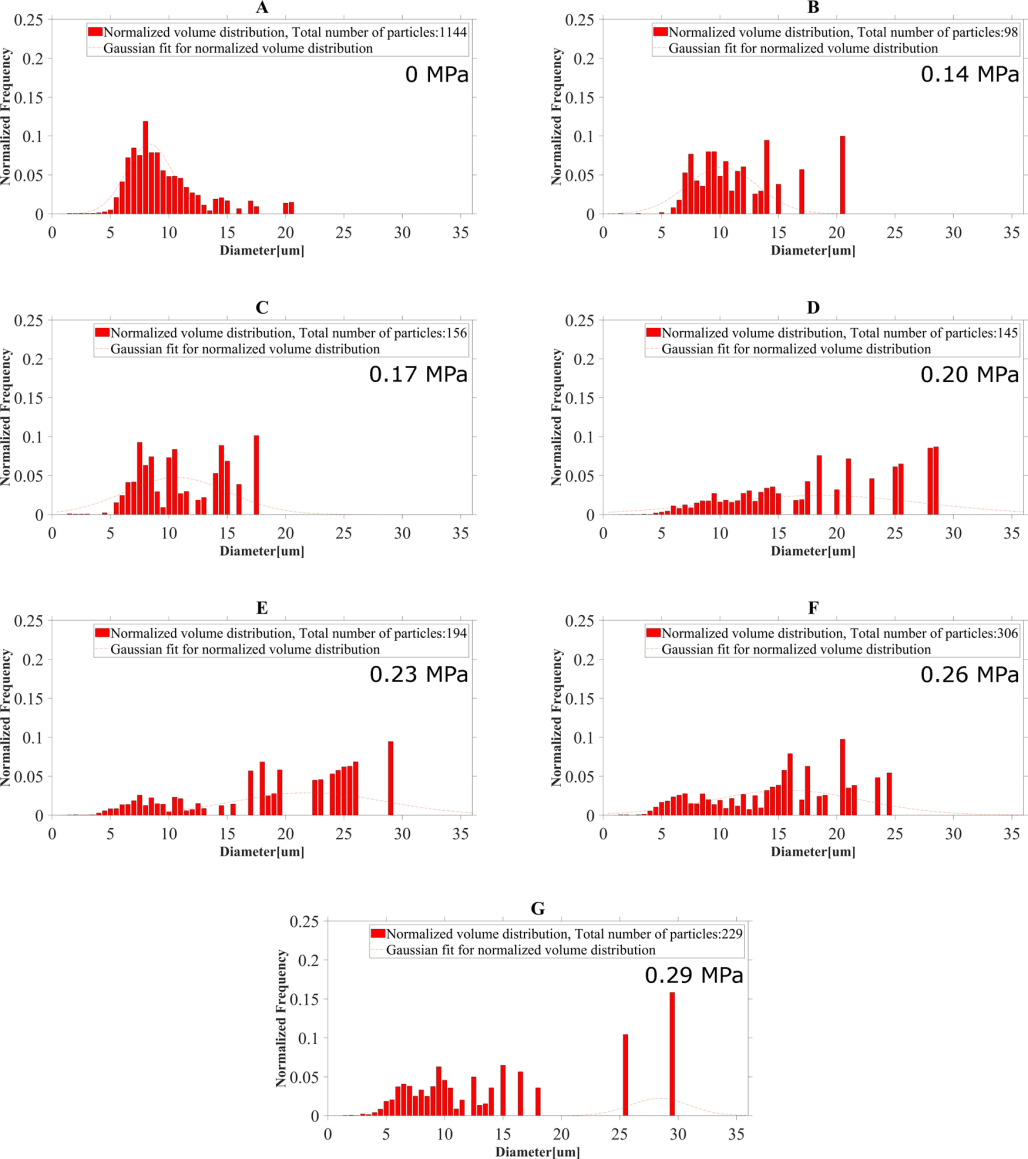
Volume size distribution for PRF of 500 Hz and burst length of 12 cycles at (a) no ultrasound exposure, (b) 0.14, (c) 0.17, (d) 0.20, (e) 0.23, (f) 0.26, and (g) 0.29 MPa. No significant changes in volume distribution of the microdroplets could be observed at pressures up to 0.17 MPa. The median value did not change as the pressure increased to 0.20 MPa and higher; however, there was a considerable increase in the number of bubbles with a larger diameter, suggesting that larger droplets underwent ADV.

The volume distribution of the microdroplets at different acoustic pressures is presented as histograms in Figure 6. The median value at each PNP did not change significantly as the pressure increased as well as the interquartile range. At 0.20 MPa, a significant increase in the maximum diameter was observed, and the standard deviation of the volume distribution became much larger compared to PNP below 0.20 MPa. As the number of cycles in the excitation pulse decreased from 12 to 4, the maximum observed diameter decreased as well, although the threshold still was 0.20 MPa. Decreasing the pulse repetition frequency (PRF) from 500 to 100 Hz resulted in no visible change in the volume distribution at any used PNP, suggesting that no ADV took place.

### Imaging Signature of the Acoustic Response in a Confined Medium

An imaging setup was developed to visualize the acoustic response of the droplets at various PNPs and number of cycles in the release pulse. Higher acoustic pressures were utilized as PNP below 0.20 MPa showed no acoustic response. The influence of the number of cycles was tested by changing the value to 1, 8, 64, and 256 at a constant PNP of 1.67 MPa, and the change of mean pixel value was compared in between all of the trials. The results presented in Figure 8 show that as the number of cycles in the release pulse increases, so does the rate of decrease in the mean pixel value in the region of interest (ROI). When 1 cycle is used, the decrease was steady and almost linear, while at 256 cycles, the decrease in mean pixel value was steeper but was only observed after the first three sequences; there was barely any change in the mean pixel value after that. This indicated that a longer release pulse led to a more destructive behavior. It is worth noting that when the flow was restarted in between measurements, less droplets recirculated in the system. One explanation for this might be that the same sample was used for all the experiment run. Therefore, it is possible that the destruction of the droplets in previous experiments had a negative impact on the droplet concentration.

**Figure 7:**
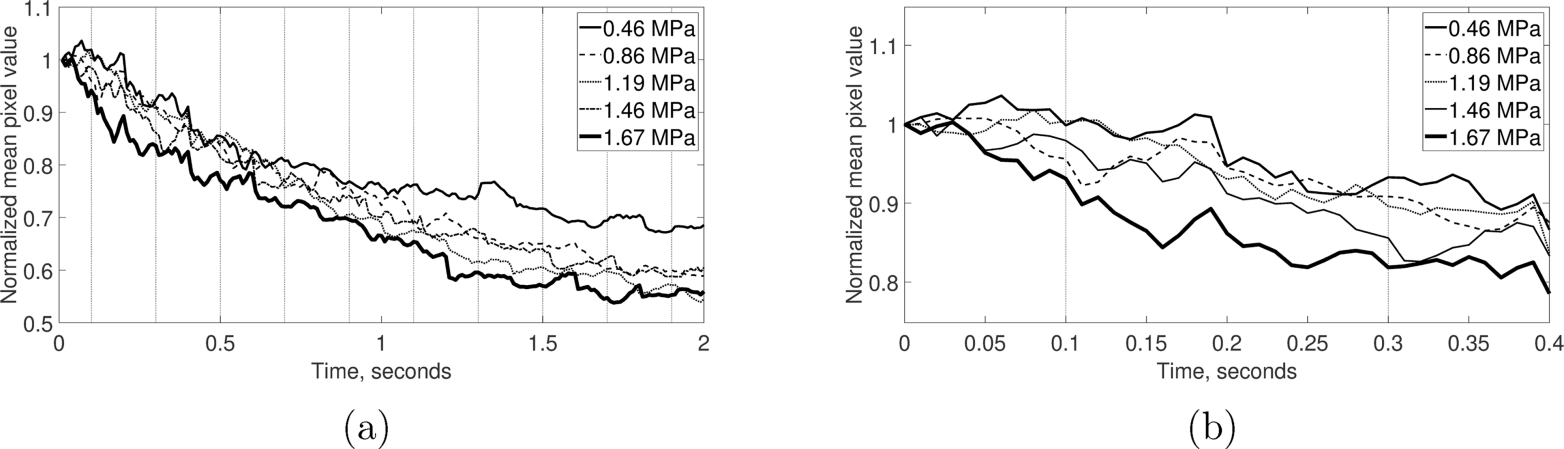
Normalized pixel value inside the ROI given at a PNP of 0.46–.67 MPa for (a) all 10 image-release-image sequences and (b) first two image-release-image sequences. Ten images with 10 ms in between were taken both before and after each release pulse, marked with vertical lines. Data was normalized with the initial value at *t* = 0. The lowest and highest acoustic pressures are marked with blue and pink lines, respectively. The vertical lines show the time points at which the release pulse was transmitted.

To compare the effects of the different acoustic pressures, we first normalized the mean pixel values inside the vessel for each experiment. The plots of these datasets are presented in Figure 7.

At 0.46 MPa, after the 10 consecutive image-release-image pulse sequences (or 2 s), about a third of the initial intensity value was lost as indicated by Figure 7. At 0.02 s, intensity increased above the initial value. This increase in intensity indicated a stronger echo in the ROI, consistent with the formation of gas microbubbles. This was also observed at 1.4 s. In general, the mean pixel intensity of the ROI, however, decreased, suggesting either displacement of the gas microbubbles or their destruction.

At 1.67 MPa, these intensity spikes are mainly not present, except after delivery of the third sequence. After 10 sequences, about half of the initial intensity value was lost, indicating a more destructive behavior than at lower PNP. The high intensity pulse and long release pulse duration thus appeared to burst the droplets.

## Discussion

The current study presents the physical response of CNF-stabilized PFC5 droplets to ultrasound in a bulk and confined medium as well as an imaging setup with an L7-4 transducer employed to capture the acoustic response of these droplets at various PNPs. The combination of PFC5 and CNF enables an increased stability,^6,24^ and the manufacturing method enables a higher production rate, a comparatively narrow volume distribution, and a simple and straightforward way^9^ compared to other techniques, such as those involving microfluidics.^27^

PFC5 has a boiling temperature of 29 °C at atmospheric pressure. A pressure called the Laplace pressure, which is the pressure present due to the presence of surface tension at the oil/water interface and depends on the size of the droplets, provides additional thermal stability to the droplets by elevating the boiling temperature of the PFC5 core. Ghorbani et al.^9^ showed that the volume distribution of similar particle-stabilized droplets changed significantly between 4 and 45 °C with an increasing number of smaller bubbles present and a general broadening of the peak. We did not take thermal effects into consideration in this study as all measurements were done at room temperature, ranging from 20 to 25 °C. However, there is always a risk to induce local heating when using ultrasound even in clinical settings, especially after ADV as microbubbles require less acoustic energies to cause heating compared to liquids.^28^

The vaporization threshold for a liquid-filled droplet depends on many parameters. Among other factors, the vaporization threshold is inversely proportional to droplet diameter^5,27^ as discussed in several studies.^13,17^ It can be concluded that it is easier to vaporize larger droplets rather than smaller ones. The fact that the proportion of larger droplets has increased while the proportion of smaller droplets has stayed similar throughout the measurements suggests that some of the CNF-shelled droplets underwent ADV. This is demonstrated in both Figures 5 and 6, suggesting that ADV took place at PNP values above 200 kPa when the PRF was 500 Hz. The ADV threshold was therefore concluded to be close to 0.46 MPa as demonstrated in the confined medium, imaging system case, where a pixel intensity increase could be observed at the lowest PNP. Previous studies have shown that decreasing the burst length and decreasing the PRF increase the ADV threshold,^27,29^ which is in line with our findings.

**Figure 8:**
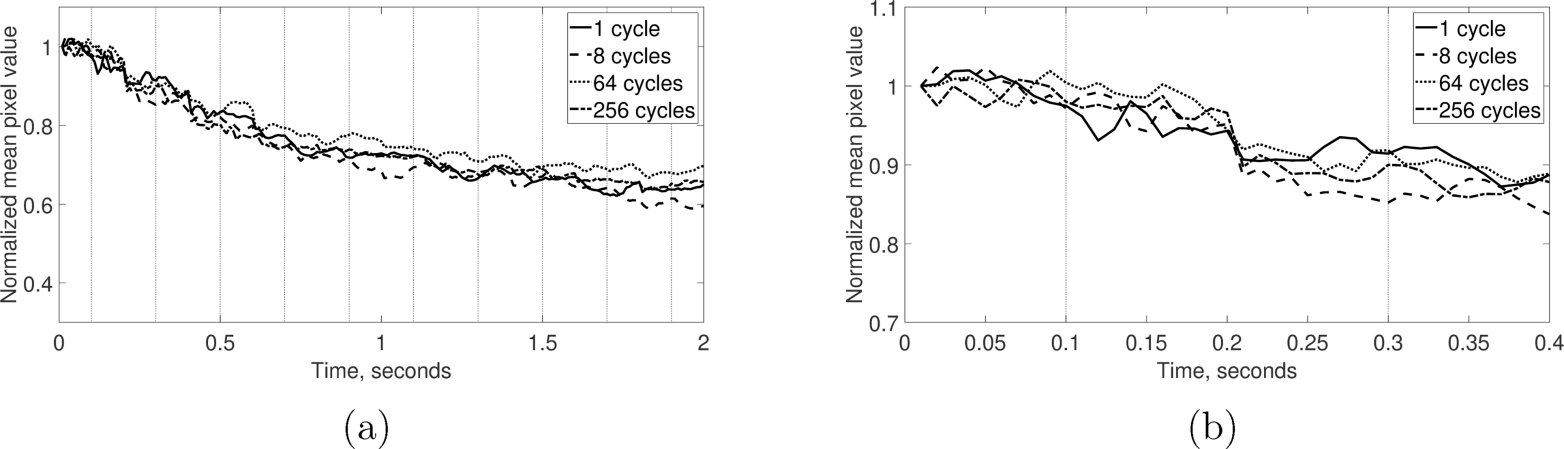
Normalized pixel value inside the ROI for 1, 8, 64, and 256 number of cycles for the release pulse. Ten images with 10 ms in between were taken before and after each release pulse, marked with black vertical lines. Data was normalized with the initial value at *t* = 0. The two graphs show (a) all 10 image-release-image sequences and (b) first two image-release-image sequences. No flow was present in the channel during each sequence.

The imaging setup developed for these particle-stabilized droplets successfully visualized vaporized bubbles by using pulse inversion imaging. The rationale to why pulse inversion was used is that only droplets that have been converted to microbubbles will be detected and not liquid-filled droplets. When comparing Figures 7 and 8, it becomes evident that the effect of PNP is greater than the effect of number of cycles in the excitation pulse. As PNP increases above the vaporization threshold, the droplet response becomes more non-linear, and at even higher values, destruction occurs.^15^ Studies have shown that a shorter pulse gives rise to higher vaporization thresholds.^30^ Reversely, a higher pulse repetition frequency results in an increase in the acoustic response.^29^ In general, a longer time window will result in a higher probability of vaporization taking place.^5^ The results obtained were in line with previous studies. For example, Puett et al.^31^ showed that the microbubble cloud size was significantly larger when a maximum pulse length of 15 cycles was used compared to the minimum pulse length of 2 cycles only. However, the steepest increase is when the pulse length changes from 2 to 5 cycles per pulse. Just as for the confined medium–single crystal setup, the pulse length has a significant role in the acoustic response of the droplets.

The results obtained from the ADV in the bulk and confined volumes reveal the significance of the investigation of ultrasound imaging with the aid of PCCA in a realistic microenvironment and open media. Several parameters like the viscosity and surface tension of the surrounding medium may reduce the efficiency of the ADV process in the confined medium; however, the comparison on diameter expansion of the PFC5 droplets between these volumes shows that there is no significant difference, which could be due to the lower boiling temperature of the droplets at the atmospheric pressure. It is of great importance as the variations that are the heat transfer and dissipation in the confinement are also among the parameters that affect the ADV process. Therefore, while it was expected to have substantial decrease in the properties of the ADV in the confined geometries, i.e., microvessel confinement, the proposed droplets could be an alternative to be used in these kinds of media. It should be noted that more investigation regarding the oscillating amplitude and signal magnitude for these droplets are required to study the contrast enhancement in the confined media.

The inertial cavitation threshold of similar perfluorocarbon droplets and their feasibility in high-intensity focused ultrasound-mediated clot lysis was studied by Pajek et al.^20^ The threshold did not change significantly with droplet size for droplets ranging from 136 to 552 nm, which was in agreement with the results present in this study. Through inertial cavitation, it is also possible to further enhance the uptake of drug in tumor tissues.^32^ Therefore, the CNF-coated droplets have the potential to be used as drug carriers in ultrasound-mediated drug delivery. However, in order to use it for medical applications, it is necessary to understand how these droplets interact with living matter. The smallest capillaries in the human body have a diameter of approximately 10 μm,^5^ which therefore must be the upper limit for droplet sizes in order not to cause unwanted blockage. Additionally, a protein corona might form around the droplets when placed in biological materials, causing a change in the chemical properties of microdroplets.^33^ Further studies regarding the bioeffects of these droplets are therefore required.

## Conclusions

A new class of particle-stabilized perfluoropentane droplets has been tested, which demonstrate acoustic droplet vaporization (ADV) at different levels of acoustic power in no flow conditions. First, we tested the droplets in a bulk medium to find their properties in ideal conditions, then in a confined medium, and lastly, using a programmable imaging system to test the effect of pulse duration and excitation pressure on the ADV threshold.

Evidence pointing toward ADV was found for two different combinations of ultrasound properties for an unfocused transducer in the confined medium. For both cases a significant increase in the standard deviation of the volume distribution could be seen for peak negative pressure (PNP) amplitudes of 0.20 MPa. The sudden increase in the standard deviation indicated that ADV has occurred and that the pressure threshold for ADV is located between 0.20 and 0.23 MPa for ultrasound waves with these properties. For higher PNP values, the ultrasound had a reduced effect on the volume distribution possibly due to the higher pressure leading to the destruction of droplets and release of their inner content. The vaporization threshold was the same at different burst lengths; however, the extent of ADV was larger when the number of cycles was higher.

An acoustic pressure of 0.45 MPa showed to be sufficient to visualize the ADV effect using an imaging setup. The signal intensity decreased for all excitation pressures between 0.45 and 1.67 MPa due to an increasing non-linear response and finally destruction of the vaporized bubbles. As these values were within medically approved limits (MI = 0.89 at 1.67 MPa), the present results successfully demonstrate the potential of these droplets as a phase-changing contrast agent. More studies are undertaken to show their potential for clinical ultrasound-mediated applications.

## Materials and Methods

#### Materials

Bleached sulfite pulp, kindly donated by Nordic Paper Seffle AB, Säffle, Sweden, was used for the production of CNFs. The preparation of cationic CNFs, a quaternary ammonium salt modified CNF with 0.13 mmol cationic groups per g of fiber, is described elsewhere.^24^ The CNFs were ca. 4 nm in width and of varying lengths up to the micrometer range. PFC5 (99%) was purchased from Apollo Scientific (Stockport, U.K.). EcoSolv A, 99.5% (Solveco Chemicals AB, Rosersberg, Sweden) was purchased for cleaning of tubing by flushing after each measurement.

#### Droplet Fabrication

The CNF-stabilized droplets were produced according to a scheme described elsewhere.^9,24^ In short, a 0.303 wt % CNF suspension (pH = 7.46) was first obtained by diluting a stock suspension (1.3 wt % solid content) in water by sonication using a 1/2 in. tip and Sonics Vibracell W750 (Sonics and Materials Inc., Newton, CT, US) at 80% amplitude for 1 min. Then, the diluted CNF suspension was mixed with PFC5 using the same sonicator. A schematic of the procedure is shown in Figure 1.

The volume distribution of the droplets was assessed by light microscopy (Eclipse Ni-E, Nikon Corporation, Japan) and dynamic light scattering (ZetaSizer Nano ZS, Malvern Panalytical, UK) measurements.

The droplets had a concentration of 4.7 × 10^7^ ml^–1^. For the experiments, the solution was diluted in Milli-Q water with a 1:19 dilution ratio.

#### Acoustic Tests in Ideal Conditions

CNF-stabilized PFC5 droplets (100 μL) were added to 1900 μL of deionized water in order to prepare the final mixture, which was exposed to the ultrasound waves. Droplets were introduced into the sample chamber (Opticell, Thermo Fischer Scientific, U.S.) equipped with optically and acoustically transparent walls. The sample chamber was immersed in a water bath for efficient acoustic coupling. The acoustic tests were performed using a high-power tone burst pulser-receiver (SNAP Mark IV, Ritec, Inc., Warwick, RI, USA) equipped with a flat transducer (V382-SU Olympus NDT, Waltham, MA) operating at a single frequency of 3.5 MHz. The emulsion of CNF-stabilized PFC5 droplets was exposed to the acoustic pressure that varied between 0.04 and 0.29 MPa at the given frequency to a stationary suspension. To investigate volume variations in vaporized droplets at each pressure level, they were first visualized using an upright transmitted light microscope (ECLIPSE Ci-S, Nikon, Tokyo, Japan) equipped with a 20× magnification objective, and then the diameter of the vaporized droplets was measured with the aid of the ImageJ software (version 1.50b, National institutes of health, USA) to determine the concentration and volume distribution. Only vaporized droplets that were floating on the surface or were in the vicinity of the top of the measurement container were investigated. This was done in order to ensure that the studied vaporized droplets were gas filled. An in-house image edge detection MATLAB script (MathWorks Inc., Natick, MA) was applied to analyze the images obtained from the microscope and to provide the volume distributions. The Gaussian distribution accompanied the plot with the mean value and standard deviation calculated from the experimental data.

#### Effect of Confined Volume on the Acoustic Response

The first series of confined medium–single crystal experiments were performed to study the effects of the confined medium on ADV at the same levels of acoustic pressures as in the bulk medium described previously. The setup consisted of a water bath with an unfocused transducer (V382-SU Olympus NDT, Waltham, MA) integrated into the bottom of the tank, plastic tubing (Zeus Inc., Orangeburg, SC, USA), a holder, and a syringe (BD Plastipak 1 mL, Becton Dickinson, Franklin Lakes, NJ, USA). The transducer was powered by a high-power tone burst pulser-receiver (SNAP Mark IV, Ritec, Inc., Warwick, RI, USA) as shown in Figure 2.

The setup was used to expose the droplets to ultrasound with a frequency of 3.5 MHz and a pulse repetition frequency (PRF) of either 100 or 500 Hz. The number of cycles in each pulse was set to 4, 8, or 12 at a PRF of 500 Hz and to 12 at a PRF of 100 Hz. A number of acoustic parameters were changed in between runs in an attempt to characterize the ADV process of the droplets. The polytetrafluoroethylene tubing (Zeus, Inc., Orangeburg, SC, US)^34^ was inserted into the holder, which was then submerged into the water bath and placed in the focal region of the transducer. The tubing had an inner diameter of 500 μm and a wall thickness of 50 μm to resemble the size of a small arteriole.^34^ The PFC5-suspension was gently mixed by hand, and then the syringe was used to withdraw the suspension. The syringe was then taped to the side of the water bath and connected to the plastic tube via a needle. The syringe was used to push the suspension through the tube with a flow rate of 10 μL/s. The ultrasound was activated when there was no flow present in the tubing. The other end of the tube was then placed over a microscopy slide (VWR International, Radnor, PA, USA), and the suspension was allowed to drop from the end of the tube onto the slide after ultrasound exposure. The slide was placed under the microscope after suspension deposition, and images of the droplets were obtained.

With the ultrasound activated, the attenuation of the electrical output was set to −10 dB using an attenuator (RA-31 Ritec, Inc., Warwick, RI, US)^34^ and then decreased to 0 dB in intervals of 2 dB, corresponding to PNPs of 0.14 to 0.29 MPa, respectively. Finally, the second batch of reference images was taken after exposing the suspension to ultrasound.

#### Imaging Signature of the Acoustic Response in a Confined Medium

The setup consisted of a focused linear array transducer (ATL L7-4, Philips, Netherlands) in combination with an ultrasound system (Verasonics V-1 Research System, Verasonics Inc., Kirkland WA, USA), a tissue mimicking phantom (Peripheral Vascular Doppler Flow Phantom, Model 524, ATS Laboratories, USA), and droplets made with a highly cationic CNF as described previously. The 8 mm vessel in the tissue phantom was filled with droplets diluted in water, while the 6 mm vessel was filled with distilled water as the reference.

Two sets of experiments were performed. The first experiment aimed to investigate the dependence of the ADV threshold on the ultrasound pulse duration. Thus, the number of cycles in the pulse was varied from 1 to 8, 64, and 256 cycles while keeping the same excitation pressure of 1.67 MPa. The second experiment aimed to investigate the dependence of the ADV threshold on the excitation pressure while keeping the pulse duration constant and equal to its maximum value of 384 cycles. For that experiment, five release pressure values of 0.46, 0.86, 1.19, 1.46, and 1.67 MPa were employed. The pressure of the imaging pulse was the same in both experiments and equal to 0.46 MPa, i.e., the smallest value considered in the study of 0.46 MPa. This pulse sequence was called an image-release-image pulse sequence. Ten consecutive images with a time interval of 10 ms were captured before and after each release pulse. The number of cycles of the imaging pulse was set to 2. All of the above measurements were initiated when there was no flow present in the vessel.

Pulse inversion images were taken in all experiments with a transmitted frequency of 3.5 MHz and a PRF of 500 Hz. The frequency and PRF were held constant during each measurement. For each image obtained, three ROIs of similar sizes were selected as depicted in Figure 3. Two of these regions marked with blue and green were in the tissue part of the tissue mimicking phantom, while the third one, the red, was in the vessel containing the droplets. For each ROI, the intensity value of each individual pixel was added together and divided by the number of pixels to obtain the mean pixel intensity in linear space.
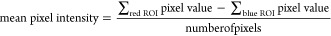
1

Additionally, the mean pixel values inside the vessel were normalized in regards to the initial mean pixel value for each experiment.

## Supporting Information Available

(Figure S1) Volume distribution of CNF-shelled droplets at a PRF of 100 Hz and no ultrasound, 0.14, 0.17, 0.20, 0.23, 0.26, and 0.29 MPa; (Figure S2) one of the original images taken after ultrasound exposure in the polytetrafluoroethylene at a PRF of 500 Hz, a PNP of 0.29 MPa, and 8 cycles with image taken at the bottom of the Bürker chamber, where the droplets were in focus and the image is taken in another visual plane to show the big gas microbubble located at another focus plane than the non-vaporized droplets; and (Section S3) pressure calibration: method section explaining how the pressure calibration was performed throughout this study (PDF)

## References

[ref1] Gramiak R.; Shah P. M. Echocardiography of the Aortic Root. Invest. Radiol. 1968, 3, 356–366. 10.1097/00004424-196809000-00011. 5688346

[ref2] Ziskin M. C.; Bonakdarpour A.; Weinstein D. P.; Lynch P. R. Contrast agents for diagnostic ultrasound. Invest. Radiol. 1972, 7, 500–505. 10.1097/00004424-197211000-00006. 4644894

[ref3] Cosgrove D.; Harvey C. Clinical Uses of Microbubbles in Diagnosis and Treatment. Med. Biol. Eng. Comput. 2009, 47, 813–826. 10.1007/s11517-009-0434-3. 19205774

[ref4] Bokor D. Diagnostic Efficacy of SonoVue. Am. J. Cardiol. 2000, 86, 19–24. 10.1016/S0002-9149(00)00985-1. 10997347

[ref5] Zhou Y. Application of Acoustic Droplet Vaporization in Ultrasound Therapy. J. Ther. Ultrasound 2015, 3, 1–18. 10.1186/s40349-015-0041-8. 26566442 PMC4642755

[ref6] Sheeran P. S.; Matsuura N.; Borden M. A.; Williams R.; Matsunaga T. O.; Burns P. N.; Dayton P. A. Methods of Generating Submicrometer Phase-Shift Perfluorocarbon Droplets for Applications in Medical Ultrasonography. IEEE Trans. Ultrason. Ferroelectr. Freq. Control 2016, 64, 252–263. 10.1109/TUFFC.2016.2619685. 27775902 PMC5706463

[ref7] Sirsi S. R.; Borden M. A. State-of-the-Art Materials for Ultrasound-Triggered Drug delivery. Adv. Drug Delivery Rev. 2014, 72, 3–14. 10.1016/j.addr.2013.12.010. PMC4041842 24389162

[ref8] Giesecke T.; Hynynen K. Ultrasound-mediated cavitation thresholds of liquid perfluorocarbon droplets in vitro. Ultrasound in medicine & biology 2003, 29, 1359–1365. 10.1016/S0301-5629(03)00980-3. 14553814

[ref9] Ghorbani M.; Olofsson K.; Benjamins J.-W.; Loskutova K.; Paulraj T.; Wiklund M.; Grishenkov D.; Svagan A. J. Unravelling the Acoustic and Thermal Responses of Perfluorocarbon Liquid Droplets Stabilized with Cellulose Nanofibers. Langmuir 2019, 35, 13090–13099. 10.1021/acs.langmuir.9b02132. 31549511

[ref10] Schad K. C.; Hynynen K. In vitro characterization of perfluorocarbon droplets for focused ultrasound therapy. Physics in Medicine & Biology 2010, 55, 4933 10.1088/0031-9155/55/17/004. 20693614

[ref11] Kang S.-T.; Lin Y.-C.; Yeh C.-K. Mechanical Bioeffects of Acoustic Droplet Vaporization in Vessel-Mimicking Phantoms. Ultrason. Sonochem. 2014, 21, 1866–1874. 10.1016/j.ultsonch.2014.03.007. 24690297

[ref12] Lin S.; Zhang G.; Jamburidze A.; Chee M.; Leow C. H.; Garbin V.; Tang M.-X. Imaging of Vaporised Sub-Micron Phase Change Contrast Agents with High Frame Rate Ultrasound and Optics. Phys. Med. Biol. 2018, 63, 065002 10.1088/1361-6560/aaac05. 29384498

[ref13] Ho Y.-J.; Yeh C.-K. Theranostic Performance of Acoustic Nanodroplet Vaporization-Generated Bubbles in Tumor Intertissue. Theranostics 2017, 7, 1477 10.7150/thno.19099. 28529631 PMC5436507

[ref14] Kripfgans O. D.; Zhang M.; Fabiilli M. L.; Carson P. L.; Padilla F.; Swanson S. D.; Mougenot C.; Brian Fowlkes J.; Mougenot C. Acceleration of Ultrasound Thermal Therapy by Patterned Acoustic Droplet Vaporization. J. Acoust. Soc. Am. 2014, 135, 537–544. 10.1121/1.4828832. 24437794 PMC3985868

[ref15] Loskutova K.; Grishenkov D.; Ghorbani M. Review on Acoustic Droplet Vaporization in Ultrasound Diagnostics and Therapeutics. BioMed Res. Int. 2019, 2019, 1 10.1155/2019/9480193. PMC6662494 31392217

[ref16] Rehman T. U.; Khirallah J.; Demirel E.; Howell J.; Vlaisavljevich E.; Yuksel Durmaz Y. Development of Acoustically Active Nanocones Using the Host–Guest Interaction as a New Histotripsy Agent. ACS Omega 2019, 4, 4176–4184. 10.1021/acsomega.8b02922. 31459627 PMC6649115

[ref17] Kripfgans O. D.; Fowlkes J. B.; Miller D. L.; Eldevik O. P.; Carson P. L. Acoustic Droplet Vaporization for Therapeutic and Diagnostic Applications. Ultrasound Med. Biol. 2000, 26, 1177–1189. 10.1016/S0301-5629(00)00262-3. 11053753

[ref18] Radhakrishnan K.; Holland C. K.; Haworth K. J. Scavenging Dissolved Oxygen via Acoustic Droplet Vporization. Ultrason. Sonochem. 2016, 31, 394–403. 10.1016/j.ultsonch.2016.01.019. 26964964 PMC4788814

[ref19] Yildirim A.; Shi D.; Roy S.; Blum N. T.; Chattaraj R.; Cha J. N.; Goodwin A. P. Nanoparticle-Mediated Acoustic Cavitation Enables High Intensity Focused Ultrasound Ablation Without Tissue Heating. ACS Appl. Mater. Interfaces 2018, 10, 36786–36795. 10.1021/acsami.8b15368. 30339360 PMC6702128

[ref20] Pajek D.; Burgess A.; Huang Y.; Hynynen K. High-Intensity Focused Ultrasound Sonothrombolysis: The Use of Perfluorocarbon Droplets to Achieve Clot Lysis at Reduced Acoustic Power. Ultrasound Med. Biol. 2014, 40, 2151–2161. 10.1016/j.ultrasmedbio.2014.03.026. 25023095 PMC4130783

[ref21] Li D. S.; Kripfgans O. D.; Fabiilli M. L.; Brian Fowlkes J.; Bull J. L. Initial Nucleation Site Formation due to Acoustic Droplet Vaporization. Appl. Phys. Lett. 2014, 104, 063703 10.1063/1.4864110. 24711671 PMC3970834

[ref22] Jin Q.; Lin C.-Y.; Kang S.-T.; Chang Y.-C.; Zheng H.; Yang C.-M.; Yeh C.-K. Superhydrophobic Silica Nanoparticles as Ultrasound Contrast Agents. Ultrason. Sonochem. 2017, 36, 262–269. 10.1016/j.ultsonch.2016.12.001. 28069209

[ref23] Stocco A.; Drenckhan W.; Rio E.; Langevin D.; Binks B. P. Particle-Stabilised Foams: An Interfacial Study. Soft Matter 2009, 5, 2215–2222. 10.1039/b901180c.

[ref24] Svagan A. J.; Benjamins J.-W.; Al-Ansari Z.; Shalom D. B.; Müllertz A.; Wågberg L.; Löbmann K. Solid Cellulose Nanofiber Based Foams–Towards Facile Design of Sustained Drug Delivery Systems. J. Controlled Release 2016, 244, 74–82. 10.1016/j.jconrel.2016.11.009. 27847327

[ref25] Lin S.; Zhang G.; Leow C. H.; Tang M.-X. Effects of microchannel confinement on acoustic vaporisation of ultrasound phase change contrast agents. Phys. Med. Biol. 2017, 62, 6884 10.1088/1361-6560/aa8076. 28718774

[ref26] Svagan A. J.; Musyanovych A.; Kappl M.; Bernhardt M.; Glasser G.; Wohnhaas C.; Berglund L. A.; Risbo J.; Landfester K. Cellulose Nanofiber/Nanocrystal Reinforced Capsules: A Fast and Facile Approach Toward Assembly of Liquid-Core Capsules with High Mechanical Stability. Biomacromolecules 2014, 15, 1852–1859. 10.1021/bm500232h. 24716647

[ref27] Lea-Banks H.; O’Reilly M. A.; Hynynen K. Ultrasound-Responsive Droplets for Therapy: A Review. J. Controlled Release 2019, 293, 144–154. 10.1016/j.jconrel.2018.11.028. PMC6459400 30503398

[ref28] Moyer L. C.; Timbie K. F.; Sheeran P. S.; Price R. J.; Miller G. W.; Dayton P. A. High-Intensity Focused Ultrasound Ablation Enhancement In Vivo via Phase-Shift Nanodroplets Compared to Microbubbles. J. Ther. Ultrasound 2015, 3, 7 10.1186/s40349-015-0029-4. 26045964 PMC4455327

[ref29] Seda R.; Harmon J.; Fowlkes J. B.; Bull J. Use of Pulse Repetition Frequency to Augment Acoustic Droplet Vaporization In Vivo. J. Acoust. Soc. Am. 2016, 140, 3026–3026. 10.1121/1.4969388.

[ref30] Lo A.; Kripfgans O.; Carson P.; Rothman E.; Fowlkes J. Acoustic droplet vaporization threshold: effects of pulse duration and contrast agent. IEEE Trans. Ultrason. Ferroelectr. Freq. Control 2007, 54, 933–946. 10.1109/TUFFC.2007.339. 17523558

[ref31] Puett C.; Sheeran P. S.; Rojas J. D.; Dayton P. A. Pulse Sequences for Uniform Perfluorocarbon Droplet Vaporization and Ultrasound Imaging. Ultrasonics 2014, 54, 2024–2033. 10.1016/j.ultras.2014.05.013. 24965563

[ref32] Gao Z.; Kennedy A. M.; Christensen D. A.; Rapoport N. Y. Drug-Loaded Nano/Microbubbles For Combining Ultrasonography and Targeted Chemotherapy. Ultrasonics 2008, 48, 260–270. 10.1016/j.ultras.2007.11.002. 18096196 PMC2637393

[ref33] Tenzer S.; et al. Rapid Formation of Plasma Protein Corona Critically Affects Nanoparticle Pathophysiology. Nat. Nanotechnol. 2013, 8, 772–781. 10.1038/nnano.2013.181. 24056901

[ref34] Grishenkov D.; Kari L.; Brodin L.-Å.; Brismar T. B.; Paradossi G. In Vitro Contrast-Enhanced Ultrasound Measurements of Capillary Microcirculation: Comparison between Polymer-and Phospholipid-Shelled Microbubbles. Ultrasonics 2011, 51, 40–48. 10.1016/j.ultras.2010.05.006. 20542310

