# Supplementary material for: A Study on the Acoustic Response of Pickering Perfluoropentane Droplets in Different Media
Source: ACS Omega. 2021 Feb 17;6(8):5670–8. doi: 10.1021/acsomega.0c06115 (PMC7931408; doi:10.1021/acsomega.0c06115)
Supplement: Supplementary file 1 — ao0c06115_si_001.pdf [file ao0c06115_si_001.pdf]

A Study on the Acoustic Response of Pickering Perfluoropentane Droplets in Different Media

Ksenia Loskutova,[*,†] Didrik Nimander,[†] Isabelle Gouwy,[†] Hongjian Chen,[†] Morteza Ghorbani,[†,‡] Anna J. Svagan,[¶] and Dmitry Grishenkov[†]

†*Department of Biomedical Engineering and Health Systems, KTH Royal Institute of Technology, Stockholm, Sweden*

‡*Sabanci University Nanotechnology Research and Application Center, Istanbul, Turkey*

¶*Department of Fibre and Polymer Technology, KTH Royal Institute of Technology, Stockholm, Sweden*

E-mail: ksenial@kth.se

Phone: +46 (0)707 267677

S1 Effect of confined medium on the acoustic response at PRF of 100 Hz

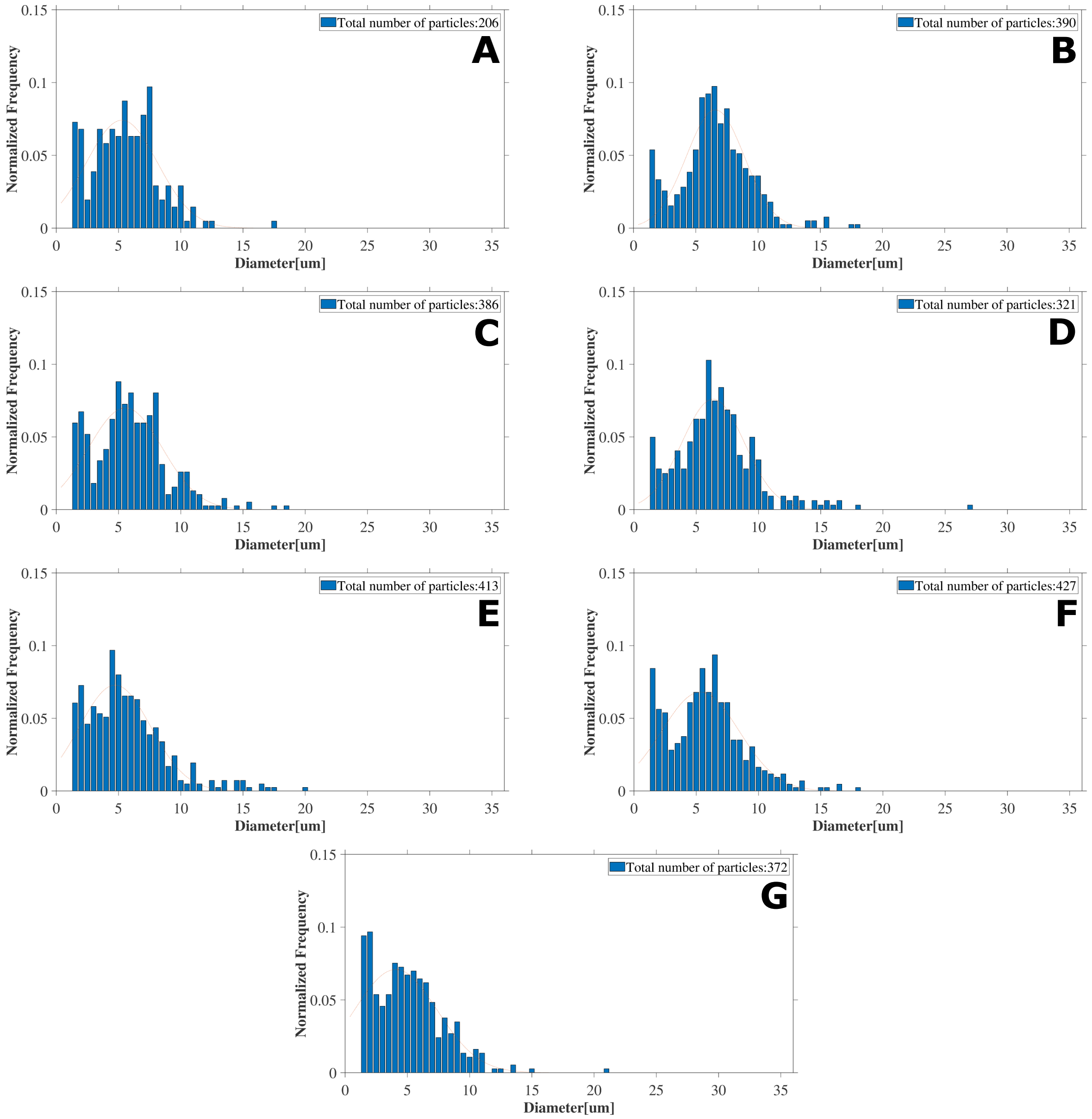

Figure S1: The volume distribution of CNF-shelled droplets at a PRF of 100 Hz and a) no ultrasound, b) 0.14 MPa, c) 0.17 MPa, d) 0.20 MPa, e) 0.23 MPa, f) 0.26 MPa, g) 0.29 MPa.

S2 Original files after ultrasound exposure in confined medium

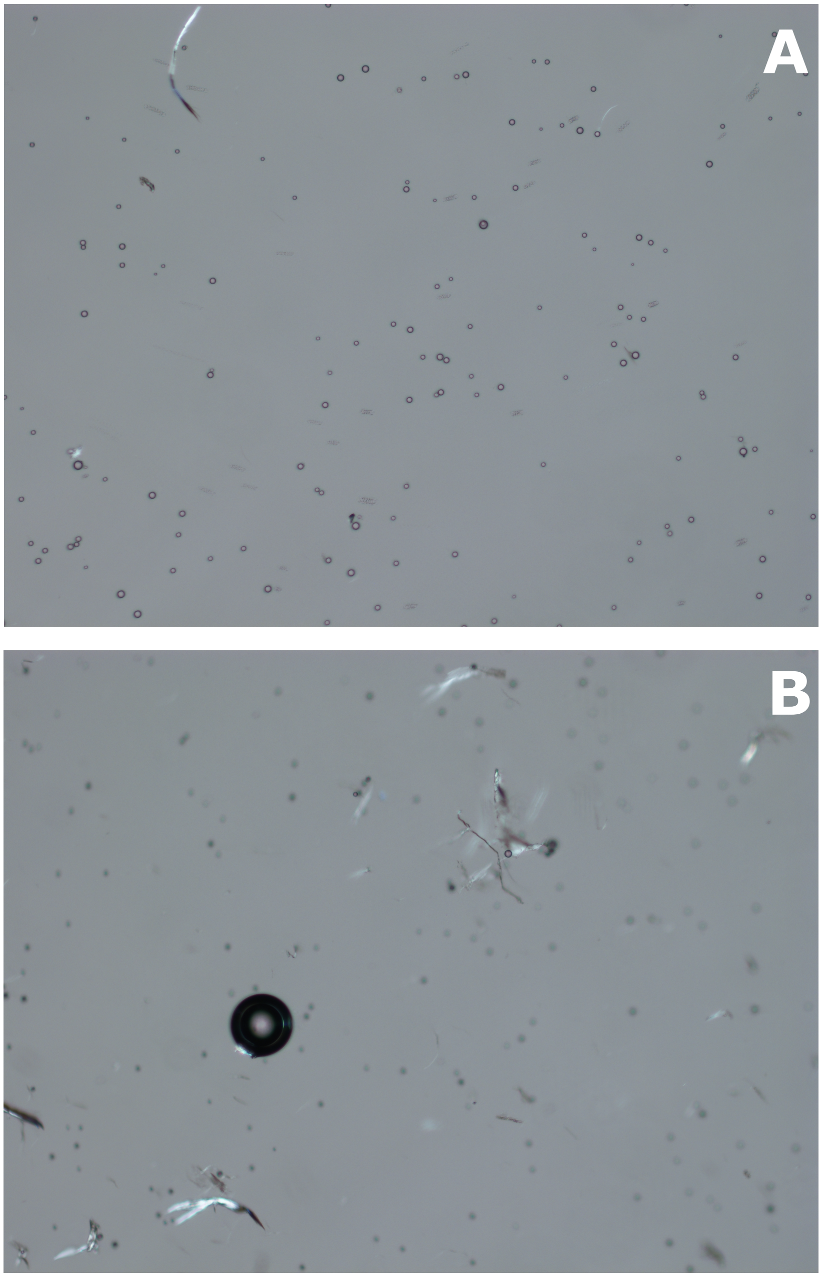

Figure S2: Two of the original images, taken after ultrasound exposure in the polytetrafluoroethylene at a PRF of 500 Hz, a PNP of 0.29 MPa and 8 cycles; a) image taken at the bottom of the Burker chamber, where the droplets were in focus; b) the image is taken in another visual plane to show the big gas microbubble located at another focus plane than the non-vaporized droplets. The photos were taken by the authors.

S3 Pressure calibration

The PNP at the focused points for all parts of the study were estimated following the Equation ,

$$P_{\mathbf{e}} = \frac{P_{\mathbf{w}}}{10^{\alpha df/20}} \tag{1}$$

where $P_{\mathbf{e}}$ is the estimated PNP, $P_{\mathbf{w}}$ is the PNP obtained in water at same depth as focused point, α is the attenuation coefficient of the material, d is the distance between the transducer and the focused point, and f is the transmission frequency of the ultrasound.

PNP of the transmitted ultrasound wave at respective peak-to-peak voltage of both the transducer L7-4 (linear array L7-4, Advanced Technology Laboratories, Philips, WA, USA) and single crystal was obtained by an Acoustic Intensity Measurement System (AIMS III, ONDA, CA, USA). The transducer was fastened to the system so that it sent ultrasound wave vertically down within a water tank. The transmission ultrasound wave was sent out repetitively following a trigger signal. The trigger signal was used to synchronize and control movement of a hydrophone (HMB-200, ONDA, CA, USA) immersed in the water tank aiming to scanning the PNP of a desired transmission ultrasound wave in a 3D volume.

$P_{\mathbf{w}}$ was obtained in the calibration experiment of L7-4. d is the focus depth 17.4mm. α is the acoustic attenuation coefficient of the phantom which is 0.5 dB/cmâĹŹMHz. The transmission frequency f was set to 3.5 MHz.

For the single crystal, an electrical attenuation of 16, 10, 8, 6, 4, 2 and 0 dB at 3.5 MHz corresponded approximately to 0.09, 0.14, 0.17, 0.20, 0.23, 0.26 and 0.29 MPa, respectively. For the L7-4 transducer 10, 20, 30, 40 and 50 V at 3.5 MHz of transmitted frequency corresponded to 0.45, 0.86, 1.19, 1.46 and 1.67 MPa, respectively. The procedure and values for L7-4 transducer are the same as in the study performed by Chen et al.[1]

References

(1) Chen, H.; Evangelou, D.; Loskutova, K.; Ghorbani, M.; Grishenkov, D. On the Development of a Novel Contrast Pulse Sequence for Polymer-Shelled Microbubbles. *IEEE Trans. Ultrason. Ferroelectr. Freq. Control* **2020**, DOI: 10.1109/tuffc.2020.3041206.
